# Supplementary material for: Brush border intermicrovillar adhesion limits bacteria attachment to the small intestine brush border
Source: bioRxiv. 2025 Dec 31:2025.11.25.690363. Preprint. [Version 2] doi: 10.1101/2025.11.25.690363 (PMC12772999; doi:10.1101/2025.11.25.690363)
Supplement: Supplement 1 [file NIHPP2025.11.25.690363v2-supplement-1.pdf]

bioRxiv preprint doi: https://doi.org/10.1101/2025.11.25.690363; this version posted December 31, 2025. The copyright holder for this preprint (which was not certified by peer review) is the author/funder, who has granted bioRxiv a license to display the preprint in perpetuity. It is made available under aCC-BY 4.0 International license.

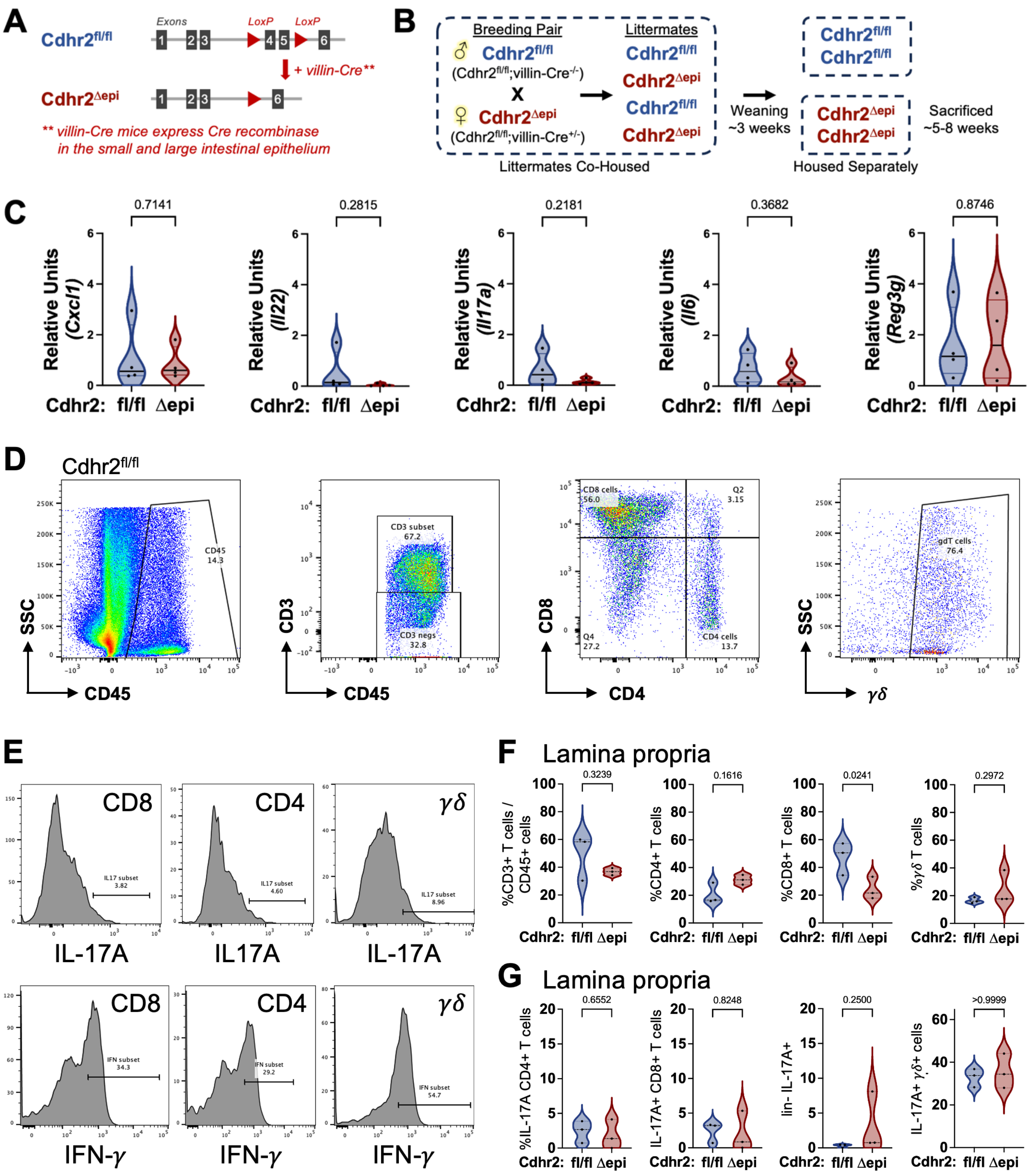

Supplemental Figure S1. Mice with a disrupted IMAC do not have increased inflammation. (A) Simplified diagram of the strategy used to generate *Cdhr2*$^{\Delta epi}$ mice from *Cdhr2*$^{fl/fl}$ in combination with tissue-specific expression of Cre; previously described in Pinette et al. 2019[30]. (B) Diagram of breeding strategy. Using a background of *Cdhr2*$^{fl/fl}$ mice, introduction of a single copy of villin-Cre (*villin-Cre*$^{+/-}$) in the mating pair results in a mixed litter of *Cdhr2*$^{fl/fl}$ and *Cdhr2*$^{\Delta epi}$

bioRxiv preprint doi: https://doi.org/10.1101/2025.11.25.690363; this version posted December 31, 2025. The copyright holder for this preprint (which was not certified by peer review) is the author/funder, who has granted bioRxiv a license to display the preprint in perpetuity. It is made available under aCC-BY 4.0 International license.

mice. (C) The expression of *Cxcl1, Il22, Il17A, Il6* and *Reg3g* in RNA extracted from the terminal ileum at 6-8 weeks of age was assessed by qRT-PCR. Relative units are calculated as described in the Materials and Methods, *Gapdh* was used as endogenous control. Statistical analysis performed using unpaired t-tests. Error bars represent mean ± SEM. (D-G) Flow cytometry analysis of T cells within the lamina propria in knockout C*dhr2*$^{\Delta epi}$ and control *Cdhr2*$^{fl/fl}$ mice. (D) Two-dimensional flow cytometry plots show the gating strategy of $CD45^+$ immune cells in gastrointestinal tissue to identify $CD3^+$ T cells within $CD45^+$ immune cells, $CD4^+$ and $CD8^+$ T cells and $\gamma\delta$ T cells. (E) Histograms show gating strategy used to identify IL-17A and IFN-γ expressing cells in $CD8^+$ T cells, $CD4^+$ T cells, and $\gamma\delta$ T cells. (F) Violin plots show proportions of T cell populations in the lamina propria of matched *Cdhr2*$^{fl/fl}$ and *Cdhr2*$^{\Delta \mathrm{epi}}$ mice ($CD3^+$ T cells over total $CD45^+$ T cells) and ($CD4^+$ T cells, $CD8^+$ T cells and $\gamma\delta$ T cells over total $CD3^+$ T cells). (G) Violin plots show proportions of IL-17A expressing T cells (CD4, CD8), lineage negative and $\gamma\delta$ T cells, in the small intestine lamina propria of matched *Cdhr2*$^{fl/fl}$ and *Cdhr2*$^{\Delta epi}$ mice, n = 3. Statistical analysis performed using paired Wilcoxon test.

bioRxiv preprint doi: https://doi.org/10.1101/2025.11.25.690363; this version posted December 31, 2025. The copyright holder for this preprint (which was not certified by peer review) is the author/funder, who has granted bioRxiv a license to display the preprint in perpetuity. It is made available under aCC-BY 4.0 International license.

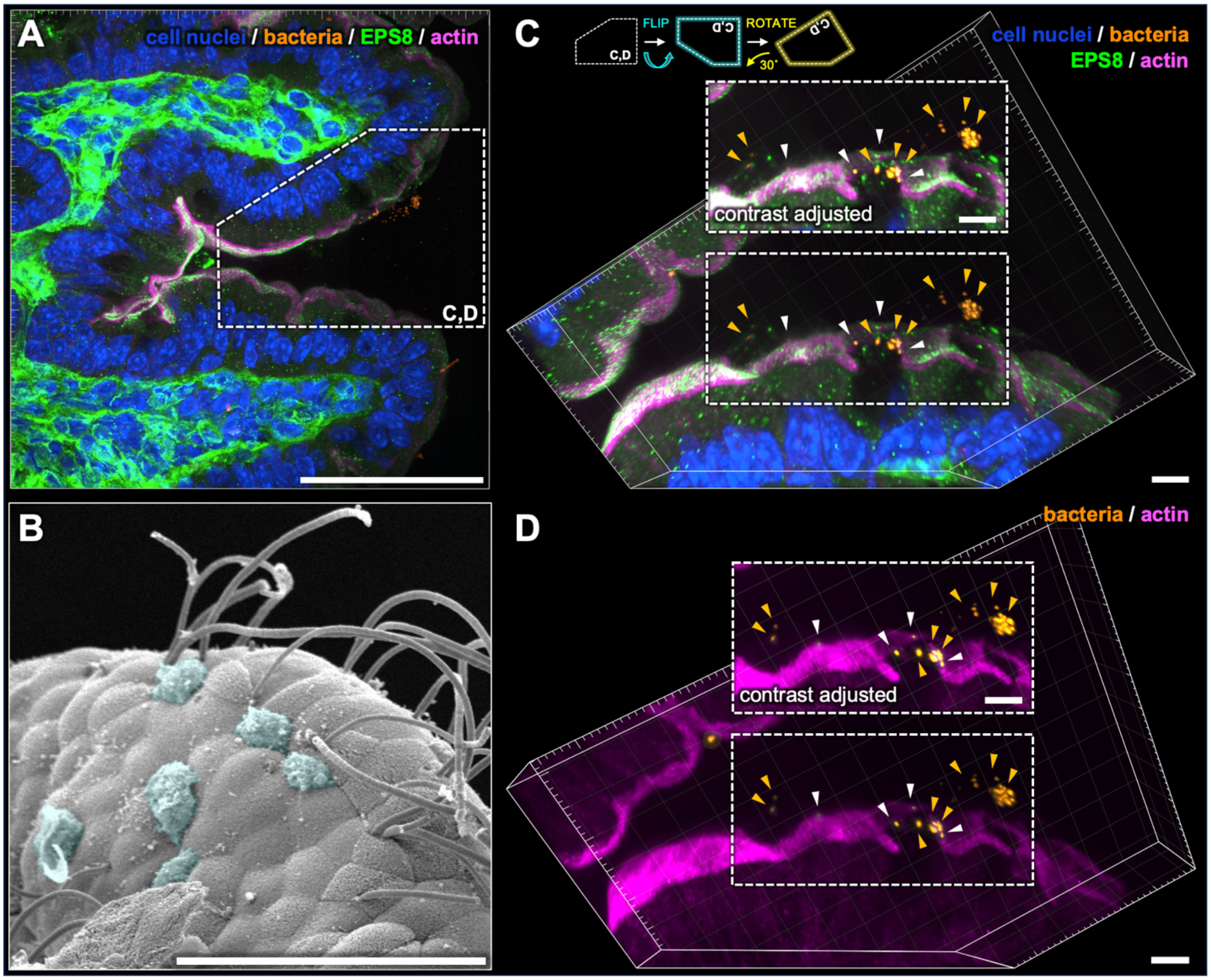

Supplemental Figure S2. Tissue-associated bacteria form actin-independent surface attachments—goblet cell and mucus binding. (A) Deconvolved confocal 3D image of *Cdhr2*$^{\Delta epi}$ ileum viewed as a maxIP (depth 11.25 μm) stained for DNA (DAPI), EPS8, and actin. DAPI segmented using an AI algorithm to isolate and differentially contrast bacteria (orange) relative to cell nuclei (blue). Boxed area enlarged and rotated in C and D. (B) SEM of *Cdhr2*$^{\Delta epi}$ ileum. Retained mucus highlighted by transparent cyan overlay. (C) Boxed area from (A) with cell nuclei, bacteria, EPS8, and actin; cropped, enlarged, contrast enhanced, and positioned (flipped vertically, rotated ~30˚ counterclockwise, tilted) to optimize visualization of bacteria relative to the cell surface. Arrowheads highlight bacteria in contact with goblet cells (white) or the adjacent lumen (orange). Boxed area in shown above with augmented brightness/contrast to visualize dim signals. (D) Boxed area from (A) showing only the bacteria and actin channels; cropped, enlarged, contrast enhanced, and positioned (flipped vertically, rotated ~30˚ counterclockwise, tilted) to optimize visualization of bacteria. Arrowheads highlight bacteria in contact with goblet cells (white) or in the adjacent lumen (orange).

bioRxiv preprint doi: https://doi.org/10.1101/2025.11.25.690363; this version posted December 31, 2025. The copyright holder for this preprint (which was not certified by peer review) is the author/funder, who has granted bioRxiv a license to display the preprint in perpetuity. It is made available under aCC-BY 4.0 International license.

Boxed area in shown above with augmented brightness/contrast to visualize dim signals. Scale bars: 50 μm (A, B), 5 μm (C, D).

bioRxiv preprint doi: https://doi.org/10.1101/2025.11.25.690363; this version posted December 31, 2025. The copyright holder for this preprint (which was not certified by peer review) is the author/funder, who has granted bioRxiv a license to display the preprint in perpetuity. It is made available under aCC-BY 4.0 International license.

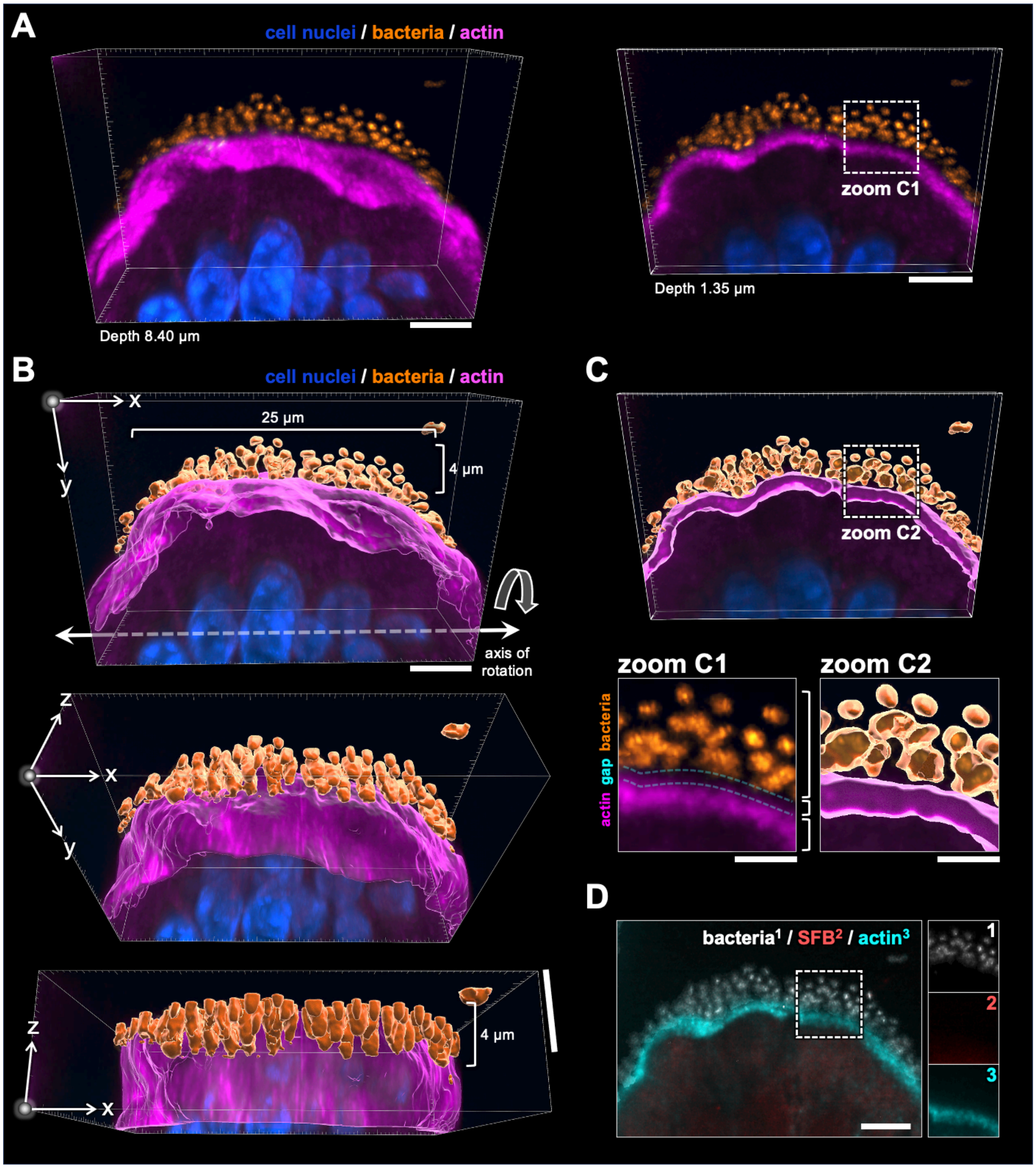

Supplemental Figure S3. Mucosal bacteria form layered three-dimensional structures that are actin-independent. (A) Villus tip from Cdhr2$^{\Delta epi}$ ileum (Figure 3J, zoom 1) viewed as a MaxIP (depth 8.40 µm, left; depth 1.35 µm, right) and rendered in 3D. Tissue stained with DAPI (DNA) and for actin. DAPI segmented using an AI algorithm to isolate and differentially contrast bacterial DNA (orange) relative to cell nuclei (blue). Boxed area enlarged in zoom C1. (B) MaxIP (depth 8.40 µm) from (A) processed using Imaris to create a 3D surface from the

bioRxiv preprint doi: https://doi.org/10.1101/2025.11.25.690363; this version posted December 31, 2025. The copyright holder for this preprint (which was not certified by peer review) is the author/funder, who has granted bioRxiv a license to display the preprint in perpetuity. It is made available under aCC-BY 4.0 International license.

fluorescent actin and bacteria DNA signals. The surface is overlaid on the fluorescence image, then sequentially rotated around the x-axis to highlight the 3D composition of the bacteria overlying the F-actin surface. (C) MaxIP (depth 1.35 μm) from viewed as a surface to highlight the relationship between the bacteria and surface actin. Boxed area enlarged and contrast enhanced in zoom C2. Zooms C1-2 show enlarged images of the original fluorescence intensity (C1) and generated surface (C2) with a clear gap between the bacteria fluorescence and surface actin and continuity of the actin surface. (D) MaxIP (depth 1.35 μm) corresponding to the image volume in (A, right) with segmented DNA (bacteria), SFB FISH (SFB), and actin. Boxed area shown as separate channels (right). Scale bars: 5 μm (A-D), 2 μm (zoom C1-2).
